# IMMUNOHISTOCHEMICAL ANALYSIS BY KI67 AND IDH1 IN PATIENTS WITH CHONDROSARCOMA

**DOI:** 10.1590/1413-785220233103e267212

**Published:** 2023-07-17

**Authors:** MARCELO BARBOSA RIBEIRO, JERÚSIA OLIVEIRA IBIAPINA, ANDRÉ MATHIAS BAPTISTA, OLAVO PIRES DE CAMARGO

**Affiliations:** 1 Universidade Federal do Piaui, Teresina, PI, Brazil.; 2 Centro Universitario Unifacid, Teresina, PI, Brazil.; 3 Associaçao Piauiense de Combate ao Cancer Alcenor Almeida, Teresina, PI, Brazil.; 4 Universidade de São Paulo, Faculdade de Medicina, Hospital das Clínicas, Instituto de Ortopedia e Traumatologia IOT HCFMUSP, São Paulo, SP, Brazil.

**Keywords:** Chondrosarcoma, Immunohistochemistry, Prognosis, Condrossarcoma, Imuno-Histoquímica, Prognóstico

## Abstract

**Objective:**

To perform an immunohistochemical evaluation using the IDH1 and Ki67 markers in patients who underwent treatment for chondrosarcoma in a reference service center in Brazil.

**Methods:**

Retrospective analytical observational study using medical records of patients diagnosed with chondrosarcoma. Besides the epidemiological and clinical profile, important variables for prognosis and correlation with immunohistochemical analysis results with Ki67 and IDH1 markers were evaluated.

**Results:**

Histopathological examinations by immunohistochemistry of 56 patients were analyzed, 52% of which were women, with the age group 20-60 years being more prevalent. Grade 1 and 2 histological subtypes corresponded to most chondrosarcomas. The femur, humerus, and tibia were the most frequent anatomical sites. Most tumors (59%) were larger than 8 cm. Ki67 expression was very low (< 10%) in 98% of patients. The analysis of IDH1 was positive in 43% of the cases. The correlation between IDH1 positivity and tumor size was statistically significant, but regarding survival, we observed no significance.

**Conclusion:**

Immunohistochemical analysis using IDH1 and Ki67 markers in patients with conventional chondrosarcoma is not useful for prognostic guidance. ***Level of Evidence II, Prognostic Assessment, Results of Immunohistochemical Tests and Correlation with Survival.***

## RESUMO

**Objetivo:**

Fazer uma avaliação imuno-histoquímica usando os marcadores IDH1 e Ki67 em pacientes que fizeram tratamento para condrossarcoma em um serviço de referência no Brasil.

**Métodos:**

Estudo retrospectivo, analítico e observacional de prontuários de pacientes com diagnóstico de condrossarcoma. Além do perfil epidemiológico e clínico, foram avaliadas variáveis importantes para o prognóstico e a correlação com os resultados da análise imuno-histoquímica utilizando os marcadores Ki67 e IDH1.

**Resultados:**

Foram analisados exames histopatológicos por imuno-histoquímica de 56 pacientes, dos quais 52% eram do sexo feminino. A faixa etária mais prevalente foi entre 20 e 60 anos. Os subtipos histológicos graus 1 e 2 corresponderam à maioria dos casos. Fêmur, úmero e tíbia foram os sítios anatômicos mais frequentes. A maioria dos tumores (59%) tinha tamanho superior a 8 cm. O Ki67 teve expressão muito baixa (< 10%) em 98% dos pacientes. Já a análise do IDH1 foi positiva em 43% dos casos. A correlação entre a positividade do IDH1 e o tamanho do tumor foi estatisticamente significativa; já em relação à sobrevida, não houve significância.

**Conclusão:**

A análise imuno-histoquímica por meio dos marcadores IDH1 e Ki67 em pacientes com condrossarcoma convencional não é útil para orientação prognóstica. **Nível de Evidência II, Avaliação Prognóstica, Resultados de Exames Imuno-Histoquímicos e Correlação com Sobrevida.**

## INTRODUCTION

Chondrosarcoma is a malignant bone tumor characterized by the formation of cartilage by tumor cells. It differs from chondroma due to its high cellularity, greater pleomorphism, and the appreciable number of pulpous cells with large or double nuclei.^1^

It presents many clinical-pathological characteristics and biological behaviors, and several distinct variants can be observed, besides the more common conventional central chondrosarcoma. They can be either primary, apparently originating from a normal bone, or secondary to some pre-existing benign cartilaginous tumor, frequently, multiple hereditary osteochondromatosis and enchondromatosis. ^(^ ^1^

According to the World Health Organization (WHO) classification in 2020, chondrosarcomas can be divided into eight subtypes. ^(^ ^2^

Type 1 - Atypical cartilaginous tumor/chondrosarcoma, grade 1;
Type 2 - Peripheral secondary chondrosarcoma;
Type 3 - Central chondrosarcoma, grades 2 and 3;
Type 4 - Secondary peripheral chondrosarcoma, grades 2 and 3;
Type 5 - Periosteal chondrosarcoma;
Type 6 - Clear cell chondrosarcoma;
Type 7 - Mesenchymal chondrosarcoma;
Type 8 - Undifferentiated chondrosarcoma.

The peak incidence of primary chondrosarcoma ranges from the fifth to seventh decades of life. Secondary chondrosarcoma affects younger individuals from the third and fourth decades of life. The clinic does not correlate with the degree or size of the lesion. ^(^ ^3^

Radiologically, the lesion is usually lithic and may contain points of intralesional calcification and cortical thickening. Histologically, it is one of the most difficult lesions for a pathologist to diagnose since the criterion for differentiating between low-grade chondrosarcoma and chondroma is uncertain. Diagnosis is based on the combination of clinical, imaging, and histopathological data. ^(^ ^4^

In most cases, biopsy only confirms it as a cartilaginous lesion. It is not entirely reliable to define the degree of malignancy of lesions *in vivo*, as the tumor may present areas with different histological grades in some cases. ^(^ ^5^

In the anatomopathological analysis, the routine staining used is hematoxylin eosin, and immunohistochemistry is rarely used. ^(^ ^5^

Treatment varies according to the histological grade, being predominantly surgical. They are tumors resistant to radiotherapy and chemotherapy, used only in high-grade cases. ^(^ ^6^

This study aimed to make a prognostic evaluation by immunohistochemistry using the markers IDH1 and Ki67 in patients who underwent treatment for chondrosarcoma.

## METHODS

The research project was registered and approved by the Ethics and Research Committee of the Hospital das Clinicas da Faculdade de Medicina da Universidade de Sao Paulo (HCFMUSP) and duly enrolled in Plataforma Brasil and by the Ethics Committee for the analysis of Research Projects (3,974,954).

Inclusion criteria: All patients with anatomopathological diagnosis of chondrosarcoma treated at the Orthopedic Oncology Service of the Instituto de Ortopedia e Traumatologia (IOT HCFMUSP) from January 2000 to December 2010. Available paraffin blocks. Clinical follow-up in 10 years.

Exclusion criteria: Medical records of patients with loss of outpatient follow-up. Single paraffin blocks in the laboratory, being unusable due to risk of loss. Only paraffin blocks from biopsies.

This was a retrospective analytical observational study using the medical records of patients diagnosed with chondrosarcoma, and “n” was used for convenience.

The following data were evaluated: period of diagnosis, age, gender, histological classification, tumor size, skeletal site, presence of metastases, distant disease-free survival in ten years, positivity for IDH1 and Ki67 immunohistochemistry.

The data were stored in a Windows Excel spreadsheet and later imported into the software.

Statistical analyses were performed using Epi Info version 7.2.5.0. The simple frequencies of all the variables studied were estimated. All were categorical and described by their count and relative frequency. Several associations were performed for statistical inference following the central object in the associations with IDH1 and Ki67. For the association between categorical variables, Pearson’s chi-square test was used, and, when appropriate, Fisher’s test or likelihood ratio test for numbers of small events observed and expected (< 10). p ≤ 0.05 was accepted as a type error for statistically significant differences.

Positivity for IDH1 (which marks the mutation of this enzyme, with a R132H clone in this study) was correlated with the anatomical site, tumor size (we used the values of < and > 8 cm according to the study by Amin et al.), ^(^ ^7^ gender, age group, histological subtype, types of surgery, systemic recurrence, and death.

The percentage of Ki67 was analyzed to standardize a prognostic score depending on statistical significance.

The analysis of IDH1 and Ki67 expression was performed by two pathologists “blindly.”

The IDH1 test was performed using the Envision flex Dako^®^ kit (K-800221) with the IDH1 antibody (clone: R132H) of the brand Gene AB^®^.

The Ki67 survey was conducted automatically, on the Dako Omnis^®^equipment.

## RESULTS

At the end of the survey of medical records, we had 83 cases, and of these, 27 were excluded, leaving 56 patients.

Regarding gender, 29% were women, the age group 20-60 years predominated with 41%, the subtypes grade 1 and 2 totaled 45% of the cases, femur and humerus were the most common anatomical sites with 56%, and tumors larger than 8 cm were the majority with 59%.

**Figure 1:**
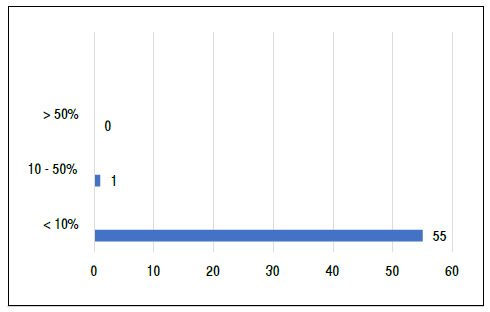
Total ki67 expression percentage.

Regarding the percentage of positivity for IDH1, 43% were obtained, whereas Ki67 was positive in only one patient (1.7%) (Figure 1).

In total, 11% of the patients had systemic recurrence.

## DISCUSSION

The current “gold standard” for the study of IDH1 mutations in chondrosarcoma is the genetic analysis of clones, cited by Amary et al., ^(^ ^8^ which is one of the limitations of this study. However, it is a high-cost method and most readily available in most reference services in Oncology. The analysis of IDH1 by immunohistochemistry is already part of the routine in several neoplasms. Positivity shows good prognosis even in tumors of the central nervous system. ^(^ ^8^

As for our “n” (used for convenience), which initially was 83 patients and after the exclusion criteria was 56, in the study of Vuong, Ngo, and Dunn, ^(^ ^9^ the casuistry of most of the selected studies can be observed and, comparing with the same period, we verified that the number of cases in our study reflects the world scenario. As well as Etchebehere et al., ^(^ ^10^ who had a similar sample comparing the periods.

The epidemiological and clinical data corroborate almost all the included studies, especially the studies by Vuong, Ngo and Dunn, ^(^ ^9^ and Etchebehere et al. ^(^ ^11^

Ki67 was suggested as a marker for analysis in our research due to its already established importance for stratification as an index of cell proliferation in sarcomas, especially in bone tumors, in which it seems to be related to biological aggressiveness and level of malignancy and may be useful in diagnosis and prognosis, such as in high-grade osteosarcoma. However, of the 56 slides, in only one the index was higher than 30% and lower than 10% in 98% of the cases, making the statistical analysis unfeasible and concluding its low expressiveness in patients with chondrosarcoma. Two studies had similar results to ours. ^(^ ^12^ ^),(^ ^13^

The low expression of Ki67 may be useful for the differential diagnosis, especially with osteosarcoma since it often presents high proliferation rates. Scotlandi et al., ^(^ ^12^ show that, in bone tumors, Ki67 seems to be related to biological aggressiveness and level of malignancy and may have a useful diagnosis and prognosis, particularly in high-grade osteosarcoma.

A genetic analysis would be necessary to assess the prognosis, according to several studies: Zhu et al., ^(^ ^14^ analyzing IDH1 mutations, concluded that they are associated with a higher relapse- and metastasis-free survival in high-grade chondrosarcomas. Lugowska et al., ^(^ ^15^ showed that IDH status would be correlated with relapse-free survival without metastases in high-grade chondrosarcomas, but the effect on overall survival requires further evaluation.

Table 1 shows that the correlation between IDH1 positivity and tumor size was statistically significant. As for the subtype (Table 2), anatomical site (Table 3), and 10-year disease-free survival (Table 4), we found no significance. Nie, Lu, and Peng, ^(^ ^16^ showed that gender, age at diagnosis, stage, grade, tumor site, surgery, and radiation are independent risk factors for survival. Amer et al., ^(^ ^17^ found that the only prognostic variable that had a significant effect on the survival of each subtype of nonconventional chondrosarcoma was metastatic disease at the time of diagnosis.

**Table 1:** Correlation between IDH1 positivity and tumor size.

IDH1	Larger than 8 cm	Smaller than 8 cm	Total
Negative	13	19	32
Positive	19	5	24
TOTAL	32	24	56

P < 0.004.

**Table 2:** Correlation between IDH1 positivity and chondrosarcoma subtype.

Chondrosarcoma subtypes						
IDH1	UD	G1	G2	G3	ME	Total
Negative	3	6	20	1	2	32
Positive	1	10	9	4	0	24
TOTAL	4	16	29	5	2	56

P = 0.108.

G: grade 1, 2, or 3; UD: undifferentiated; ME: mesenchymal.

**Table 3:** Correlation between IDH1 positivity and 10-year relapse-free survival.

10-year disease-free survival			
IDH1	Yes	No	Total
Negative	30	2	32
Positive	20	4	24
Total	50	6	56

P = 0.233.

**Table 4:** Correlation between IDH1 positivity and the anatomical site of the tumor.

Anatomical site of chondrosarcoma													
IDH1	Clavicle	Scapula	Phalanx	Femur	Fibula	Ischium	Mandible	Pelvis	Talus	Tibia	Humerus	Vertebra	Total
Negative	1	1	3	9	2	0	1	3	2	4	5	1	32
Positive	0	1	0	8	0	1	0	3	0	3	8	0	24
Total	1	2	3	17	2	1	1	6	2	7	13	1	56

P = 0.445.

**Table 5:** Correlation between anatomical site and disease-free survival in years.

	10-year disease-free survival		
Anatomical Site	YES	NO	Total
Clavicle	1	0	1
Scapula	1	1	2
Femur	14	3	17
Fibula	2	0	2
Ischium	1	0	1
Mandible	1	0	1
Pelvis	5	1	6
Talus	2	0	2
Tibia	6	1	7
Humerus	13	0	13
Vertebra	1	0	1
Total	50	6	56

P = 0.989.

**Table 6:** Frequency of the type of surgery performed.

Surgery	Frequency	Percentage
Amputation	4	7.14%
Wide resection without replacement	4	7.14%
Intralesional resection with replacement	24	42.86%
Wide resection and replacement by unconventional endoprosthesis	16	28.57%
Scapulectomy	2	3.57%
Hemipelvectomy	6	10.71%
Total	56	100.00%

**Table 7:** Correlation regarding the type of surgery and result of IDH1 analysis.

	IDH1		
Type of surgery	Negative	Positive	Total
Amputation	2	2	4
Wide resection without replacement	4	0	4
Intralesional resection with replacement	14	10	24
Wide resection and replacement by unconventional endoprosthesis	8	8	16
Scapulectomy	1	1	2
Hemipelvectomy	3	3	6
TOTAL	32	24	56

P = 0.60.

The cases with diagnosis of undifferentiated and mesenchymal chondrosarcoma were left in this study to facilitate the analysis in future studies with our series, using the genetic isolation method. Chen et al., ^(^ ^18^ identified mutations in IDH1 that were important for the differential diagnosis of undifferentiated chondrosarcoma and pleomorphic sarcoma of the bone. De Andrea, San-Julian, and Bovée, ^(^ ^19^ in an analysis of cartilaginous tumors of the bone, verified that the molecular alterations can be used for the diagnosis include alterations of IDH1 (R132C; R132H) in enchondromas, conventional chondrosarcomas, and undifferentiated chondrosarcoma. And by the HEY-NCOA2 fusion genes in mesenchymal chondrosarcoma.

The analysis of IDH1 in patients with chondrosarcoma is an essential topic, and the literature shows results that indicate the need for more prospective and comparative studies identifying factors and treatments that may influence the survival of patients with chondrosarcoma. More evidence with research could eventually lead to evidence-based treatments, avoiding the abundant exposure of patients to potentially harmful therapies, such as radiation and chemotherapy.

A greater centralization of care for patients with chondrosarcoma would be desirable and may generate opportunities for researchers to establish prospective and comparative studies.

With these results, a new analysis can be performed using clone isolation methods, multicenter studies with known metastatic cases and other statistical power analyses.

## CONCLUSION

Immunohistochemical analysis using IDH1 and Ki67 markers in patients with chondrosarcoma is not useful for prognostic guidance.
